# Innovative Imaging Techniques: A Conceptual Exploration of Multi-Modal Raman Light Sheet Microscopy

**DOI:** 10.3390/mi14091739

**Published:** 2023-09-05

**Authors:** Steffen Manser, Shaun Keck, Mario Vitacolonna, Felix Wuehler, Ruediger Rudolf, Matthias Raedle

**Affiliations:** Center for Mass Spectrometry and Optical Spectroscopy (CeMOS), University of Applied Science Mannheim, 68163 Mannheim, Germany

**Keywords:** light sheet, microscopy, Raman scattering, Rayleigh scattering, fluorescence, 3D cell cultures

## Abstract

Advances in imaging of microscopic structures are supported and complemented by adaptive visualization tools. These tools enable researchers to precisely capture and analyze complex three-dimensional structures of different kinds such as crystals, microchannels and electronic or biological material. In this contribution, we focus on 3D cell cultures. The new possibilities can play a particularly important role in biomedical research, especially here in the study of 3D cell cultures such as spheroids in the field of histology. By applying advanced imaging techniques, detailed information about the spatial arrangement and interactions between cells can be obtained. These insights help to gain a better understanding of cellular organization and function and have potential implications for the development of new therapies and drugs. In this context, this study presents a multi-modal light sheet microscope designed for the detection of elastic and inelastic light scattering, particularly Rayleigh scattering as well as the Stokes Raman effect and fluorescence for imaging purposes. By combining multiple modalities and stitching their individual results, three-dimensional objects are created combining complementary information for greater insight into spatial and molecular information. The individual components of the microscope are specifically selected to this end. Both Rayleigh and Stokes Raman scattering are inherent molecule properties and accordingly facilitate marker-free imaging. Consequently, altering influences on the sample by external factors are minimized. Furthermore, this article will give an outlook on possible future applications of the prototype microscope.

## 1. Introduction

This article presents a prototype analyzer for microscopic structures that enables 3D Raman topography and can display individual structures in a three-dimensional model. The method is based on light sheet microscopy and combines inelastic scattering of fluorescence and the Raman effect with elastic Rayleigh scattering. The system is demonstrated and validated using three-dimensional cell cultures, in particular spheroids. All measurements were carried out at the interfaculty Center for Mass Spectrometry and Optical Spectroscopy (CeMOS) of the University of Applied Sciences Mannheim.

**Figure 1:**
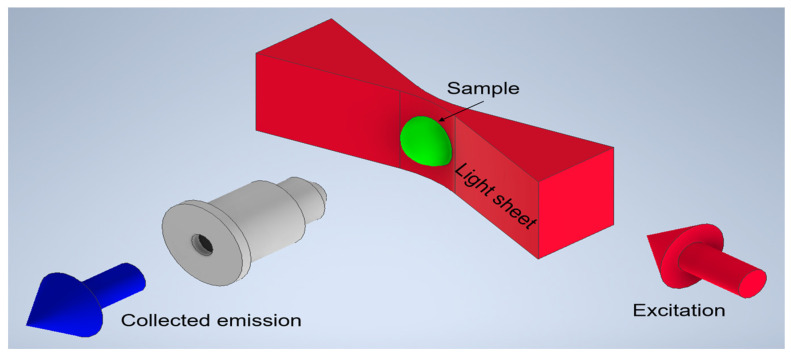
Principle of light sheet microscopy. Excitation and collection axes are orthogonally oriented with the sample placed at their intersection. A laser beam is shaped into a sheet and illuminates a thin section of the sample in the focal plane of the detection objective. The objective images the plane onto a camera chip.

There is a constant pursuit in modern biomedical research to observe and record processes on the cellular and intercellular level while minimizing any external influences on the object of interest [1,2]. Especially in the field of histology, advances are facilitated by enhancements and developments in microscopy methods [3,4]. Conventional methods of cultivating cell cultures have limited growth to virtually two spatial dimensions, effectively blocking cells from developing and interacting as they would in tissue. The development of 3D cell cultures remedied this circumstance [5,6,7]. So-called spheroids are able to grow spatially unrestricted, allowing for the formation of cell compound structures and consequently clearly differing in behavior from their conventional counterparts. This allows the creation of true tissue models for research purposes [8,9]. For instance, cell-specific markers can be tracked throughout a co-culture of distinct cell types. Another possible application is tracking the growth behavior of cancer cells in the presence of non-malignant cells. However, examining three-dimensional cell cultures requires specific methods of observation and imaging due to the increased complexity posed by, among other things, their higher thickness [10]. A suitable method is light sheet microscopy [11,12]. Herein, samples are illuminated by a laser beam shaped into a two-dimensional plane and projected into the region of interest. The illuminated plane is observed through a perpendicularly placed objective. In contrast to confocal point scanning methods, complete image planes can be photographed and stacked to create a three-dimensional model. The principle is shown schematically in Figure 1.

The axial resolution is determined by the light sheet waist, which is characterized by the beam diameter and the magnification of the illuminating objective. The detected image depends on the magnification of the imaging objective. By capturing entire planes instead of point detection, it is possible to acquire hyperspectral data quickly. Additionally, planar illumination reduces local irradiation power, thereby minimizing adverse effects and extending sample exposure time before radiation-induced damage, such as phototoxicity or photobleaching, occurs [13]. Mueller et al. (2016) described the advantages of light sheet Raman microscopy (LSRM) in the context of their developed device [14]. The authors emphasized the significantly reduced irradiance by a factor of 340 and five-fold faster sample acquisition compared to conventional confocal microscopes, which is a crucial indicator for the utilization of LSRM. They also demonstrated the applicability of LSRM to transparent samples, such as zebrafish embryos, as opaque samples can lead to reduced light penetration depth and, consequently, scattering of the measured Raman effect. The microscope presented in this article is not limited to the widely used fluorescence-based light sheet microscopy but also incorporates elastic and inelastic light scattering, specifically Rayleigh and Stokes Raman scattering [15,16]. The implementation of two illumination wavelengths (660 nm and 785 nm) allows for a high degree of flexibility in triggering multiple molecule–photon interactions. The Raman effect enables molecule-specific image contrast without the introduction of markers, minimizing external influences that may alter the sample [17]. As early as 2012, Oshima et al. demonstrated the relevance of combining light sheet microscopy and Raman spectroscopy for medical applications [18]. Furthermore, Rocha-Medoza et al. described an extension of Raman spectroscopy which combines several physical effects for a broader spectral investigation of samples [19]. This approach allows for the visualization of the sample’s natural state, in contrast to fluorescence imaging where fluorescent dyes are applied. Additionally, sample preparation is simplified and less time-consuming. Rayleigh or elastic light scattering has an effective cross-section proportional to the fourth power of the irradiating frequency [20]. This applies not only to independently scattering particles but also to higher particle concentrations, causing scattering at inhomogeneities of the refractive index. Considering the changes in the refractive index at lipid layers of cellular structures, Rayleigh scattering facilitates high spatial resolution in cell cultures, albeit without molecule-specific information [21]. Unlike Raman and fluorescence images, which provide specific information, Rayleigh scattering provides relevant morphological details of the sample, thus representing a promising approach to complement the standard LSFM method [22].

The two distinct laser wavelengths are complemented by a mechanically frictionless, tunable, high-resolution optical filter system placed in front of the camera providing specific detection capabilities over a wide spectral range. Acquired images are supplemented by spectrometric measurements. Separate imaging modalities of individual sections are overlaid, and hyperspectral images of multiple sections are then stitched to produce a 3D object. The microscope comprises all requirements for topographic mapping of molecule-selective information in 3D cell cultures.

## 2. Materials and Methods

### 2.1. Multi-Modal Light Sheet Microscope

**Figure 2:**
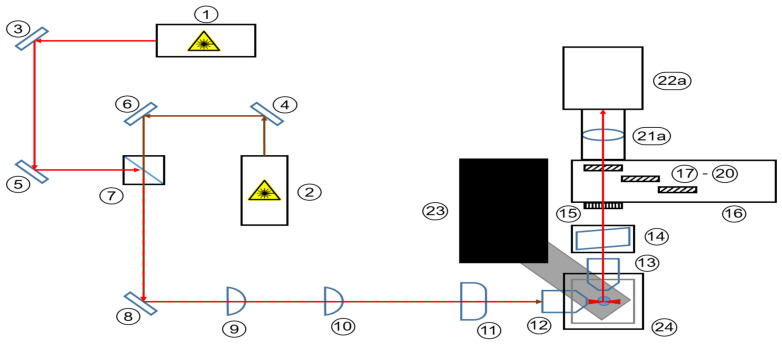
Schematic top-down view of the Raman light sheet microscope with connected sCMOS camera. 1–12: Illumination path; 13–22a: detection path; 1: Laser 1 (660 nm); 2: Laser 2 (785 nm); 3–6 and 8: broadband mirror; 7: dichroic mirror; 9: spherical lens (f = 25 mm); 10: spherical lens (f = 50 mm); 11: cylinder lens (f = 50 mm); 12: illumination objective (10×); 13: detection objective (20×); 14: AOTF; 15: polarization filter; 16: filter wheel; 17–20: longpass filters; 21a: tube lens; 22a: sCMOS camera; 23: positioning stage; 24: sample chamber. For detailed description of components, refer to Table 1.

**Figure 3:**
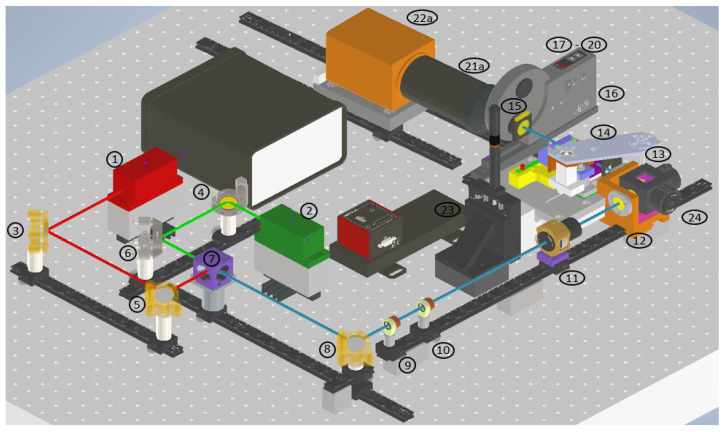
Isometric view of the Raman light sheet microscope CAD model with connected sCMOS camera. The colored lines indicate the optical path of the illuminating lasers. Red: 660 nm beam propagation. Green: 785 nm beam propagation. Blue coaxial superimposed 660 nm and 785 nm beam propagation. For detailed description of components, refer to Table 1.

The developed microscope aims at integrating multiple imaging modalities, namely Rayleigh scattering as well as Raman scattering and fluorescence emission. The sample illumination scheme is a static light sheet with orthogonal orientation of imaging and illuminating optics. The microscope is based on the OpenSPIM platform [23]. To integrate all contrast mechanisms, the setup uses a wide-range tunable spectral filter to select discreet spectral information. To initiate different photon molecule interactions, two distinct continuous wave lasers are implemented and coaxially aligned before shaping the beam cross-sections to generate static light sheets. Sample positioning then takes place in a water immersion chamber. The optical layout of the microscope is displayed as a top-down schematic and as a CAD model (Autodesk Inventor 2021) in isometric view in Figure 2 and Figure 3.

The microscope has three functional subunits: the beam-shaping and illuminating optics, the spectral selection and imaging optics and finally, at the interface of the optical units, the sample positioning mechanical unit (see Figure 2). The illuminating unit consists of both lasers, five kinematically mounted mirrors and a dichroic mirror for aligning and superimposing the laser beams, two spherical lenses and a single cylinder lens for beam shaping and, finally, a microscope objective for projecting the light sheet into the sample chamber. The five kinematic mirrors are essential for precise alignment and coaxial orientation of the laser beams. The imaging unit is comprised of a microscope objective for light detection from the illuminated sample, the acousto-optic tunable filter (AOTF) and polarization filters followed by a filter wheel containing longpass and shortpass filters and terminating in a sCMOS camera with a mounted tube lens.

Both laser beams (785 nm and 660 nm emission wavelengths) emit collimated beams with adjustable power outputs ranging from 1 to 130 mW nm and 0.5 to 200 mW for the 660 nm and 785 nm emission wavelengths, respectively. The beams are then congruently aligned on the center of the dichroic beam splitter using two mirrors, each in individually adjustable mounts oriented at 45° to the incident beam. The reflectivity of the mirrors is optimized to the respective incident lasers. At the dichroic mirror, 785 nm passes, while 685 nm is reflected. A fifth, adjustably mounted broadband mirror then relays the coaxial lasers through the center of the beam cross-section shaping optical elements. The pathway of each individual laser as well as their overlaid emission is assessed by an optical scanning slit beam profiler BP209-VIS/M (Thorlabs GmbH, Newton, NJ, USA) on a customized mount to facilitate measurement along the axis of beam propagation. After reflecting on the fifth mirror, the beam diameters are enlarged by a factor of two using two achromatic lens doublets positioned as a Keppler telescope to increase the illumination of the illuminating microscope objective. The collimated beams are then focused as a horizontal line by an achromatic cylinder lens onto the back focal plane of the illuminating 10× microscope objective. Subsequently, the illuminating objective projects the horizontal laser line as a vertical light sheet into the focal plane of the detecting microscope objective inside the sample chamber. Sample positioning is facilitated by a USB-4D-stage, which enables linear translation along the *X*-, *Y*- and *Z*-axes as well as rotation around the *Z*-axis. The linear axes have a range of 9 mm each and a resolution of 1.5 μm per step. The rotational axis has a resolution of 0.5 degrees per step. The 4D-stage is mounted on a PT1/M-Z8 single axis linear translation stage with a travel range of 25 mm and a resolution of 0.03 μm. This is performed to facilitate the possibility of sequentially imaging multiple samples by extending the travel range along the *Z*-axis. Using this combined positioning system, sample spheroids are then placed inside both the light sheet and the focal plane of the detecting microscope objective.

The light sheet itself is the intersection of the illumination and the detection optics. The detecting 20× microscope objective is orthogonally positioned toward the light sheet and collects scattered and emitted photons according to its numerical aperture. Spectral selection takes place using the subsequently placed AOTF. Here, a tellurium oxide crystal is configured as non-collinear. This causes differing propagation angles of acoustic and optic waves inside the crystal. Radio frequency waves cause the crystal to vibrate, alternating the lattice compression and relaxation and thereby varying the refractive index to only transmit desired spectral regions, effectively functioning as an adjustable bandpass filter with a wavelength-dependent bandwidth of 2.2 nm at 589 nm and 7 nm at 1060 nm [24]. The selected spectral regions are deflected at a separation angle specified by the manufacturer and dependent on the desired wavelength, relative to the 0th order. This deflection ensures that only the desired wavelength range reaches the detector. Furthermore, transmitted light is polarized. As per manufacturer recommendation, polarization filters are placed adjacent to the AOTF. Depending on the illuminating wavelength and imaging mode, further blocking filters can be placed into the beam’s path using a filter wheel. This is especially pertinent when imaging Raman scattering or fluorescence in close proximity to the exciting wavelength. Following spectral selection, a U-TLU tube lens (Evident Europe GmbH, Hamburg, Germany) images the spheroid onto the scientific complementary metal-oxide-semiconductor (sCMOS) chip of an ORCA Flash 4.0 V3 LT+ camera (Hamamatsu, Hamamatsu-shi, Japan). The camera has an effective number of 2048 × 2048 pixels at a cell size of 6.5 μm × 6.5 μm. Due to binning, the effective number of pixels is reduced to 1024 × 1024 pixels and the cell size down to 13 × 13 µm, while increasing the sensitivity of the chip by factor 4.

Lasers were selected at 785 nm and 660 nm emission wavelengths. The primary reasoning for these wavelengths is in consideration of accessing multiple molecule–photon interactions, the most critical being Raman scattering. As can be seen in Equation (1), the laser frequency, and accordingly its wavelength, is the strongest parameter when it comes to exciting the Raman effect [25]:

(1) $$I\propto \nu^{4}\cdot I_{0}\cdot N$$

**Figure 4:**
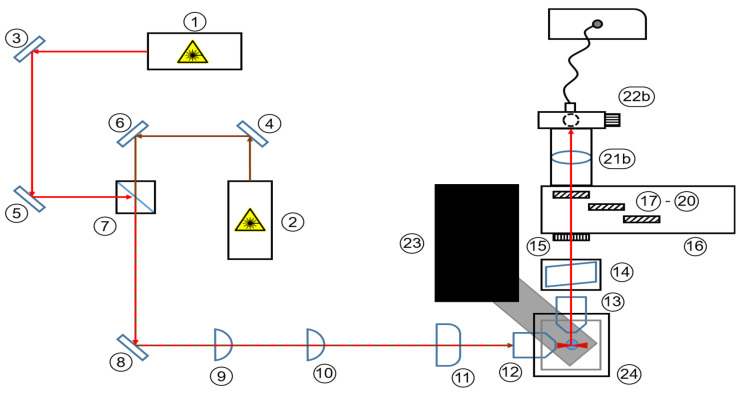
Schematic top-down view of the Raman light sheet microscope with connected spectrometer. 1: Laser 1 (660 nm); 2: Laser 2 (785 nm); 3–6 and 8: broadband mirror; 7: dichroic mirror; 9: spherical lens (f = 25 mm); 10: spherical lens (f = 50 mm); 11: cylinder lens (f = 50 mm); 12: illumination objective (10×); 13: detection objective (20×); 14: AOTF; 15: polarization filter; 16: filter wheel; 17–20: longpass filters; 21b: spherical lens (f = 25mm) with optical fiber (∅ 550 μm); 22b: spectrometer Tec5 MultiSpec^®^ Raman; 23: positioning stage; 24: sample chamber. For detailed description of components, refer to Table 1.

**Figure 5:**
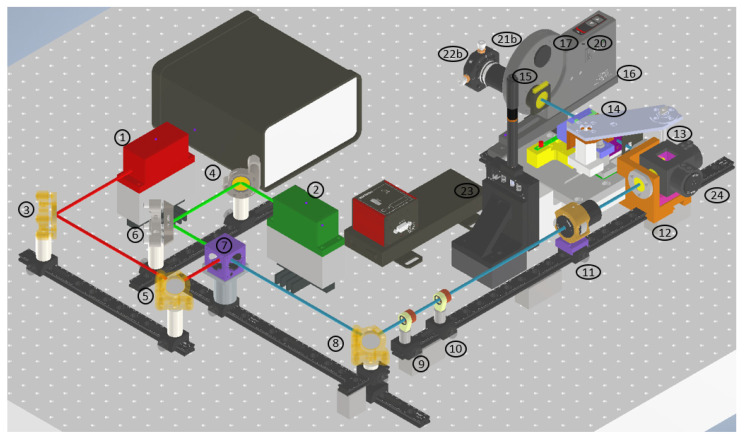
Isometric view of the Raman light sheet microscope CAD model without spectrometer. The spectrometer is not being displayed. The colored lines indicate the optical path of the illuminating lasers. Red: 660 nm beam propagation. Green: 785 nm beam propagation. Blue coaxial superimposed 660 nm and 785 nm beam propagation. For detailed description of components, refer to Table 1.

Here, we consider I for signal intensity, ν for the frequency of the molecular vibration, $I_{0}$ for the intensity of excitation radiation and N for the number of molecules in the initial state. The signal yield of inelastically scattered photons increases with decreasing wavelengths. However, it must also be taken into consideration that fluorescence is excited more efficiently, or rather more broadly, at lower wavelengths. Additionally, it should be noted that the optimal excitation wavelength for maximum fluorescence efficiency depends on the specific absorption characteristics of individual fluorophores. Therefore, it is important to consider the absorption spectrum and select the appropriate excitation wavelength accordingly. Considering this, the likelihood to overlay Raman signals with fluorescence signals increases when using lower exciting wavelengths. Given the small cross-section of the Raman effect, inelastically scattered photons become indistinguishable from fluorescence due to the mismatched signal intensities. To address both desired effects, laser wavelengths were chosen in the far red and near-infrared spectral regions. A wavelength of 785 nm is predominantly used to minimize fluorescence background in industrial Raman measurement approaches. However, when it comes to 3D cell cultures, the diversity of molecules present in the sample far surpasses any industrial process. Additionally, the molecular concentration ranges are significantly lower than in industrial applications. To address these concerns, the 660 nm wavelength guarantees a higher excitation of the Raman effect by a factor of 2 while still operating outside the optical window of tissue. A wavelength of 660 nm also gives the setup access to the fluorescent dye Draq5. To verify the spectral information of the camera, there are adjustments on the detection path. The camera is mechanically moved to a rear position, and a spectrometer adapter is attached instead. The modified setup can be seen as a top-down schematic and as a CAD model in isometric view in Figure 4 and Figure 5.

Table 1 shows an overview of the components used. The consecutive numbering corresponds to the numbers within the preceding Figure 2, Figure 3, Figure 4 and Figure 5.

**Figure 6:**
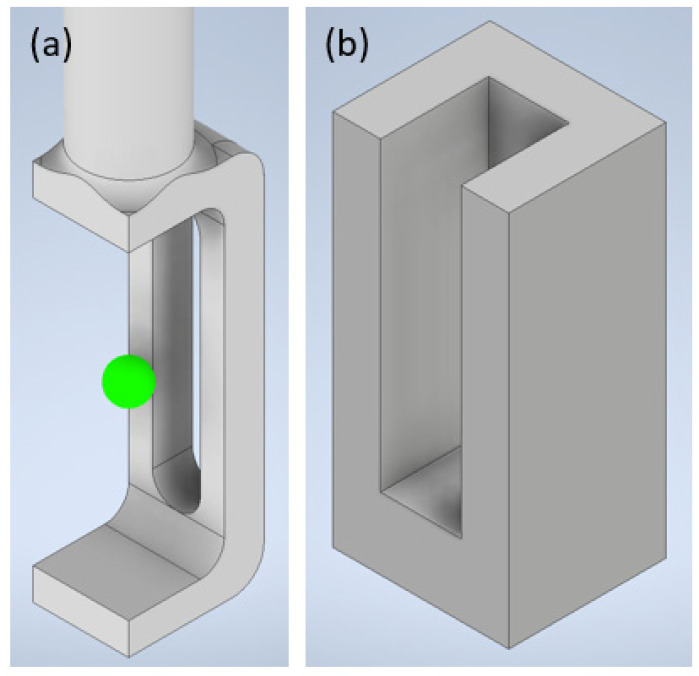
CAD model of (**a**) multi-view sample carrier and (**b**) corresponding frame for hydrogel mounting of spheroid samples.

Sample positioning was specifically designed and manufactured at CeMOS. Initial sample mounting strategies using capillaries and tubing were found lacking because of high local variability of samples when performed by different experimenters and material issues such as adhesion of spheroids to glass surfaces. To address these issues, an elongated carrier, as shown in Figure 6, was designed with a cuboidal gel chamber with a volume of 90 mm^3^ attached to a cylindrical extension, which was in turn mounted in the 4D-stage by an elastic ring. The back wall of the gel chamber was fenestrated for multi-view positioning. For sample mounting, the carrier was inserted into a corresponding frame.

Sample carriers and frames are made by 3D printing using Formlabs Form3 printers loaded with V4 black resin and are designed for single use. By switching to high-temperature V2 resin, carriers and frames are autoclavable and thereby multi-use is also possible. To further optimize sample positioning within the gel, a two-step process was developed. A negative spacer formed as an asymmetrical truncated cone is added to the frame with the carrier inserted and gel is transferred into the frame. After the gel solidifies, the result is a funnel-like gel structure terminating in the centerline of the cylindrical extension, thereby placing spheroid samples in the rotation axis of the positioning system. Spheroids are then transferred by pipette into the gel funnel and excess buffer solution is removed. Finally, the funnel structure is filled with gel to fix the spheroid in place. Inlays are designed according to the estimated diameter of spheroids.

### 2.2. Cell Culture and Spheroid Generation

To prepare spheroids in mono- and co-culture, the appropriate number of cells was seeded onto ultra-low attachment (ULA) 96-well U-bottom plates (Corning) in their corresponding medium and centrifuged at 20× *g* for 2 min. All cells were maintained in a humidified incubator at 37 °C and 5% CO_2_ fumigation and kept in culture for 3 days.

HT-29 colon cancer cells (ATCC) were cultured in McCoy’s 5A medium (Capricorn) supplemented with 10% FBS and 1% Pen/Strep. For spheroid generation, cells were detached using Trypsin/EDTA and seeded onto 96-well ULA plates at a concentration of 1 × 10^3^ cells per well. A diameter of ~300 µm was reached after three days of cultivation.

CCD-1137SK fibroblast cells derived from human foreskin (ATCC) were cultured in Iscove’s Modified Dulbecco’s Medium (IMDM, Capricorn) supplemented with 10% FBS and 1% Pen/Strep. For spheroid generation, cells were detached using Trypsin/EDTA and seeded onto 96-well ULA plates at a concentration of 2.5 × 10^3^ cells per well and cultured for three days.

For generation of co-cultures, the HT29 tumor cells were mixed 1:5 with CCD-1137SK fibroblasts. Briefly, 5 × 10^2^ tumor cells were mixed with 2.5 × 10^3^ CCD-1137SK fibroblast cells and cultured onto 96-well ULA plates for a period of three days with an equal volume of the corresponding medium.

The proof of concept consisted of 11 sequential scans per spatial orientation for spheroid analysis for a total of 33 scans, of which 27 are imaging and the remaining 6 are spectroscopic in nature. A total of 3 separate modalities were used, thereby imaging the sample at differing excitation and detection wavelengths (as described in Section 2.1) before and after fluorescence staining.

The measurements were started with manual scans mapping the boundaries of the spheroid for subsequent automated scanning. Afterwards, automated scans are carried out at a step size of 10 μm as defined by light sheet thickness.

First of all, the spheroid is embedded in 2% agarose on the application-specific carrier and suspended in the water immersion chamber. The sample positioning system, the camera as well as the AOTF are triggered by an in-house developed Python3 (version 3.7.6) program throughout the scanning procedure. In the present case, the spheroid is positioned in the center of the field of view along the *x*- and *y*-axes and then optically sectioned along the *z*-axis, while either photographs or spectra were recorded at each measurement point. Consequently, four-dimensional data (x, y, z, I) sets were acquired where x, y and z were the spatial coordinates, and I was the intensity of the detected wavelength. The step size between the optical sections matches the light sheet thickness.

Prior to each measurement, individual scan coordinates were determined specific to the investigated spheroids manually to set spatial boundary conditions for all subsequent scans. To minimize optical power exposure of cells during this, preliminary step mapping was carried out at 0.5 mW at 785 nm in camera live view. Rayleigh imaging was then carried out at 785 nm and 660 nm illumination at 1 mW power output for each wavelength and an exposure time of 100 ms. Individual sections were photographed between the border coordinates at each increment. At the end of an image stack, the spheroid is returned to the starting position. After Rayleigh data were collected, Raman stokes images were recorded. A longpass filter for the illuminating wavelength was added to the detecting light path to suppress all Rayleigh photons. Due to the low effect probability, laser power was increased to the maximum power output, and the exposure time was set to the maximum value of 5000 ms. The spheroid was then positioned at all previous sectioning planes in succession. This concluded the imaging of inherent, marker free effects. The configuration of the detecting optics was then adapted to accommodate the recording of corresponding Raman spectra of each optical section. The camera was replaced by a fiber coupling mated to a MultiSpec^®^ Raman spectrometer. To achieve a signal-to-noise ratio sufficient for peak detection, the integration time was increased to 20,000 ms. Since staining the gel-mounted spheroid with the Draq5 fluorescent marker required the sample to be removed from the measurement chamber, manual mapping followed by Rayleigh scans were repeated with the previously set parameters [26]. The last step within one set of measurements was the recording of the fluorescent-stained spheroid illuminated at 660 nm and 35 mW. The exposure time per photograph was set to 1000 ms. The detection wavelength of the AOTF was set to 815 nm, which equals 2881 cm^−1^ as central peak position. Due to the bandwidth of the AOTF of 4 nm at 815 nm, signals from 2851 cm^−1^ to 2911 cm^−1^ were detected.

The workflow of the data acquisition for one set of measurements, which includes several spectral scans, is summarized in Table 2.

### 2.3. Image Processing

Data analysis was executed separately for each modality. Photographs were stacked to create a three-dimensional model of the spheroid using the 3D Viewer plug-in for the open-source image-processing package Fiji. Before stacking took place, images were processed as necessary for the imaged spectral effect. By comparing the modalities to each other spheroid, margins were clearly identified. Following this, the image background was removed. This was performed to eliminate any objects outside the region of interest and to reduce the data volume. Due to the low signal intensity of the Raman effect, raw images of this modality appeared uniformly black. To compensate, pictures of Raman sections were processed based on their corresponding spectrometric data. Raman spectra were measured for each imaged plane. An in-house created Python script then matched each image to its spectrum. Cosmic rays that appear randomly when detectors were struck by high-energy particles were purged from raw spectra by applying an algorithm for spectra despiking. Pre-processing of Raman spectral data was completed by smoothing the Raman spectra in order to reduce noise. To that effect, a Savitzky–Golay filter with a third-order polynomial fit and a window size of 110 cm^−1^ or 7 nm was applied to the spectral region of interest [27]. The Python script then automatically identified the highest Raman peak intensity, and the pixel intensities were then rescaled according to this information. The complete spectral pre-processing as well as the intensity correction of Raman photographs was performed with the developed Python application.

## 3. Results

In this section, the results illustrating the basic operation and the technical possibilities of the presented device are presented.

### 3.1. Light Sheet Dimensions

**Figure 7:**
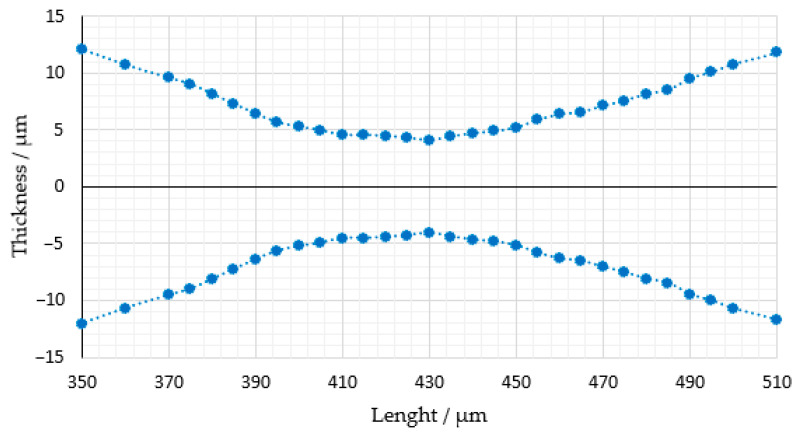
Light sheet dimensions formed by 660 nm emission wavelength. The reference point is situated at the center of the light sheet.

The axial resolution of light sheet microscopes is determined by the beam waist. Accordingly, the beam propagation of the light sheet was measured for each laser using a BP209-VIS/M scanning-slit optical beam profiler (Thorlabs). The measured dimensions are displayed in the following figures. The light sheet generated by the 660 nm laser emission is represented in Figure 7.

The measured data show a light sheet waist of approx. 8 μm resulting from the 660 nm emission, representing the minimum thickness of the sheet at this wavelength.

**Figure 8:**
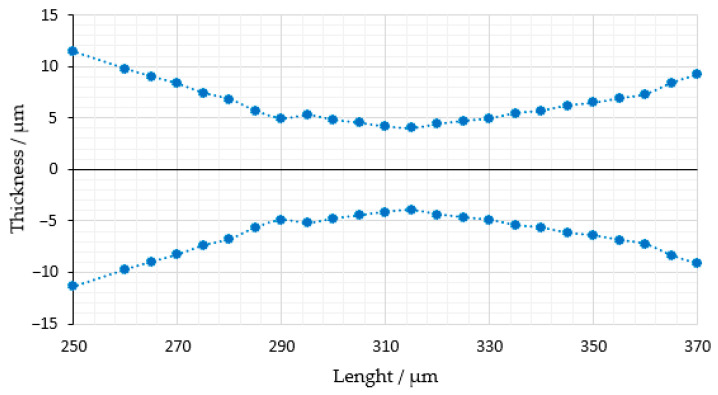
Light sheet dimensions formed by 785 nm emission wavelength. The reference point is situated at the center of the light sheet.

The beam profile data collected for the 785 nm laser emission are displayed in Figure 8.

Virtually identical to 660 nm, the emission of 785 nm forms a waist of approx. 8 μm, indicating the minimum light sheet thickness. Accordingly, the axial resolution for both lasers is 8 μm, with the minimum step size for optical sectioning set in the same order of magnitude at 10 μm. These dimensions result in a maximum planar power exposure of 7.14 mW/mm^2^.

### 3.2. Reproducability and Accuracy of the Sample Positioning System

The accuracy of the positioning system along the z- or sectioning axis is a defining factor for imaging and must be considered in regard to light sheet thickness. Increments of 10 steps are investigated, and the actual travel distance is measured with a high-precision dial gauge in μm.

The travel distance is traversed three times in the same direction to compensate for the mechanical backlash. The origin for each drive is identical for all measurements. When resetting, the origin is purposefully passed over and restarted in the direction of travel for later measurements. A mean travel distance per step is calculated from the data set. The quartiles of all measurements are displayed in Table 3.

### 3.3. Image Parameter

**Figure 9:**
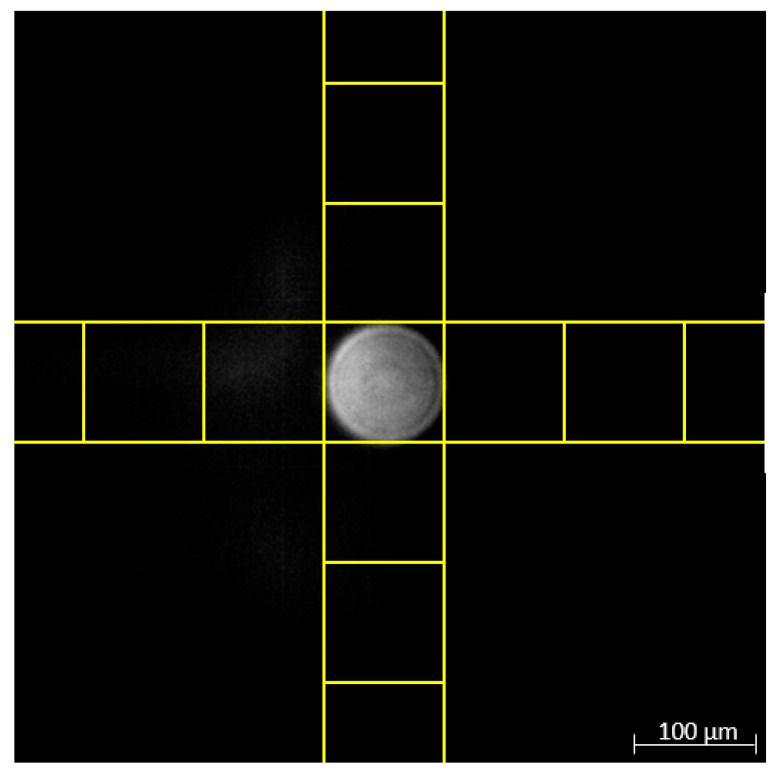
Determination of the field of view using monodisperse, spherical PMMA particles with 100 μm diameters imaged with the Rayleigh specification at 660 nm and 1 mW optical power output. Highlighted in yellow: square grid derived from the particle diameter.

In order to determine the dimensions of the field of view, monodisperse, spherical polymethylmethacrylate (PMMA) particles with a diameter of 100 μm were embedded in a 2% agarose hydrogel, illuminated at 660 nm and imaged with an exposure time of 100 ms using Rayleigh specifications for the AOTF setting. The number of pixels was counted for the maximum extent of the spherical particle. Originating at the particle, a square grid derived from the particle diameter was superimposed on the image. The results are represented in Figure 9.

The PMMA particle dimension indicates a field of view of approx. 635 μm by 635 μm for the optical components of the imaging setup. Consequently, spheroid diameters should not exceed 635 μm to facilitate imaging of complete optical sections. Throughout the experimental work, no spheroids exceeding 500 μm were investigated.

**Figure 10:**
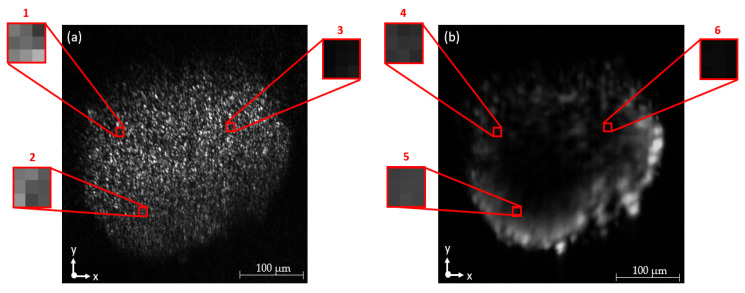
Image coordinates of investigated pixels for the determination of the signal fluctuation of the camera chip with (**a**) Rayleigh and (**b**) Raman settings. The spheroid was illuminated at 660 nm emission and detected at 670 nm with a bandwidth of 3 nm. The image was cropped from its original size to improve visibility of the examined areas.

To investigate the signal intensity variation range of imaging, two image sets of ten pictures each of a single spheroid section were taken and the pixel values compared. For one image (Rayleigh settings) set, the spheroid was illuminated with the 660 nm emission wavelength at 1 mW, and the AOTF was set to 670 nm to avoid overlaying the image with the exciting wavelength as well as eliminating signal intensity with the blocking filters. The other image set (Raman settings) was acquired at 130 mW optical power output with the AOTF set to 815 nm (ca. 2800 cm^−1^). The intensity fluctuation of individual pixels was calculated for each image set and is summarized in Table 4. In Figure 10, the investigated areas used for comparative analysis are highlighted in a sample image of the measurement set.

Table 4 shows the relative deviation of individual pixel intensities from the arithmetic mean of all investigated pixels in percent.

### 3.4. Spheroid Image Processing

**Figure 11:**
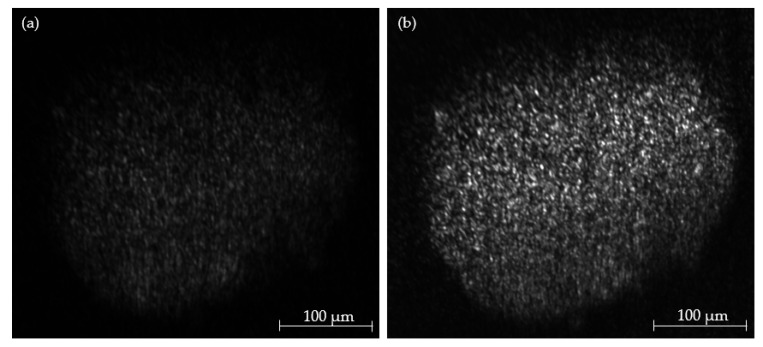
Comparison of Rayleigh images of a fibroblast HT29 spheroid illuminated at 785 nm (**a**) and 660 nm (**b**). Optical power at 785 nm and 660 nm: 7.14 mW/mm^2^. Exposure time: 100 ms. AOTF setting: 785 nm (**a**) and 660 nm (**b**), respectively.

The measurement results generated with the microscope prototype are presented in the following figures. Figure 11 shows images of a fibroblast HT29 spheroid. The spheroid was successively illuminated and imaged at 785 nm and 660 nm. Optical power output was set to 7.14 mW/mm^2^ at an exposure time of 100 ms.

Spheroids are cell cultures that have an approximately spherical shape. By utilizing Rayleigh scattering, morphological information about spheroids can be obtained. This is achieved through the analysis of the scattered light emitted at the illuminating wavelength by the spheroids. The changes in direction and intensity distribution of the scattered light can provide insights into the size, shape and internal structure of the spheroids. The results of the Rayleigh measurements show that morphological structures are visible along the entire cross-section of the spheroid for both illumination wavelengths. The imaging mode is used to determine the extent of the spheroid and to differentiate its borders from the image background. At identical optical power output and exposure time, results for 660 nm show a 40% increase in contrast compared to 785 nm calculated from the single pixel values of the pixels with the highest intensity. The signal gain comes from the wavelength-dependent higher quantum efficiency of the camera as well as the superior transmission of the tellurium oxide crystal in the AOTF.

**Figure 12:**
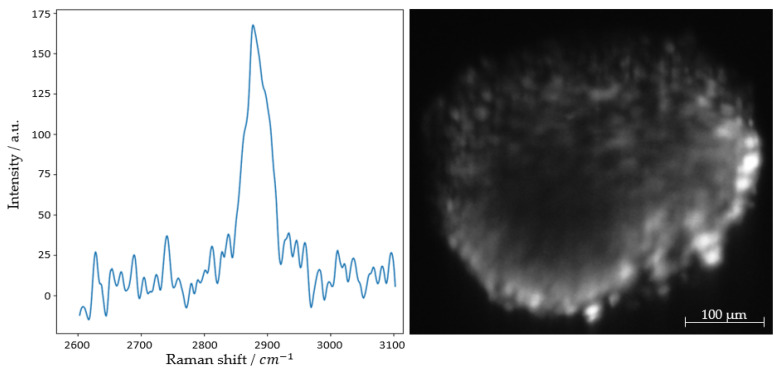
Raman spectrum and image of a fibroblast HT29 spheroid. Illuminated with 0.93 mW/mm^2^ optical power at 660 nm and detected at 815 nm or, respectively, 2881 cm^−1^ with an exposure time of 5000 ms for both the sCMOS camera and 5000 ms with the spectrometer.

Figure 12 shows the same optical section of the spheroid as the previous Figure 11 using the Raman effect. In addition to the image, the corresponding spectrum is displayed. Measurement parameters were set to 0.93 W/mm^2^ optical power output at 660 nm, detecting a central peak of 815 nm or 2881 cm^−1^ at an exposure time of 5000 ms.

Throughout the experiments, as opposed to purposefully introduced fluorescent dye, no autofluorescence was detected in the wavelength regions from 2800 to 3000 cm^−1^.

The measured spectra clearly indicate Raman scattering in the far-shifted wavenumber range, which rules out fluorescence. This is supported by the presence of characteristic hydrogen–carbon Raman signals (see Table 5).

Using the Raman effect for image contrast, single cells become discernable in the circumference of the spheroid. The size of individual cells is approx. 5 μm. The inelastically shifted Stokes photons in the signal range from 2851 cm^−1^ to 2911 cm^−1^ constitute a marker-free image. The high wavenumber region represents various molecular stretching vibrations of carbon and hydrogen bonds (see Table 5).

**Figure 13:**
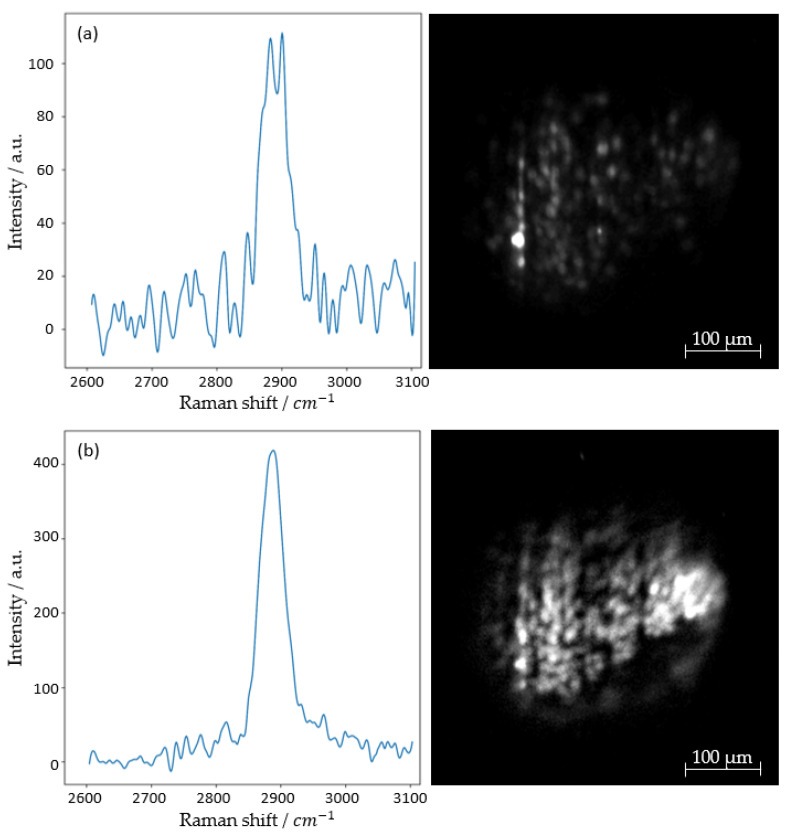
Comparison of unstained (**a**) and Draq5-stained (**b**) optical sections and respective spectral signal. The peak intensity is approx. 400% greater for stained samples.

Figure 13 compares an optical section using marker-free Raman imaging to the same spheroid after Draq5 staining. The dye Draq5 is a commonly used fluorescent stain employed in cell imaging and fluorescence microscopy [31]. It stands out for its ability to specifically stain the DNA within cell nuclei. This targeted staining allows for precise localization of cell nuclei and analysis of cell cycle and nuclear processes. Due to its reliability and applicability, Draq5 is widely adopted in biomedical research, supporting the examination of cell structures and genetic processes.

Measurement parameters were set to illumination at 660 nm and detection at 817 nm. The Raman effect was excited at 0.93 W/mm^2^ optical power output at an exposure time of 5000 ms. Fluorescence from Draq5 was excited at 0.36 W/mm^2^ at 660 nm and imaged with an exposure time of 2000 ms.

Alongside the increase in signal intensity, the use of Draq5 facilitates the reduction in optical power and the exposure time. The use of fluorescent staining, however, causes an increased effort in sample preparation.

While the spectral information seems similar, it must be considered that the emission of Draq5 spans the spectral range from 660 nm to 820 nm, effectively overlaying the majority of the Raman spectrum.

**Figure 14:**
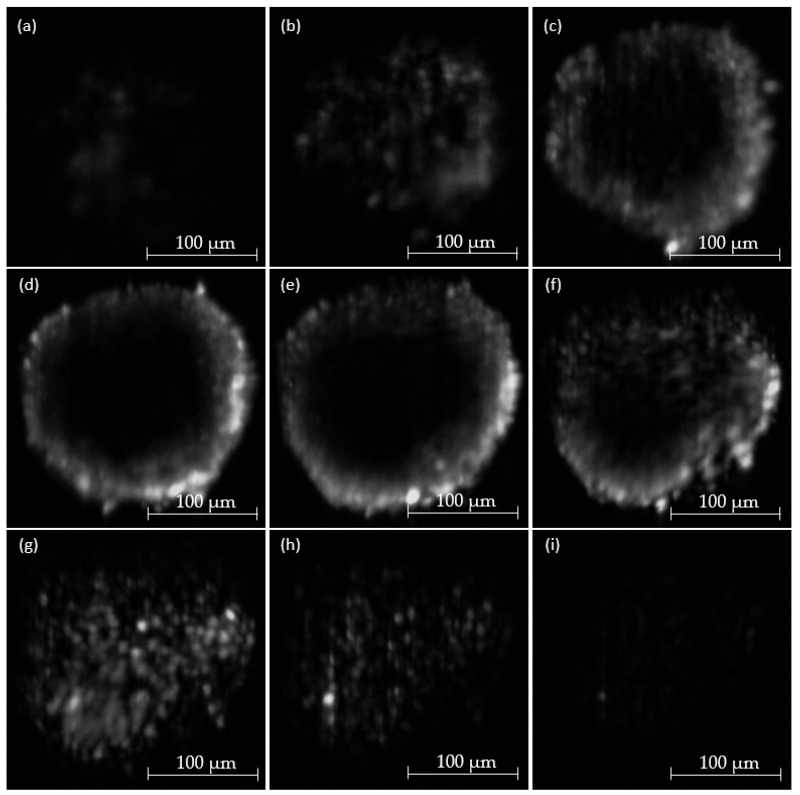
Sequence of selected optical sections of an unstained fibroblast HT29 spheroid along the *z*-axis from (**a**–**i**). Illuminating wavelength: 660 nm. Optical power output: 0.93 W/mm^2^. AOTF setting: 815 nm or 2881 cm^−1^. Exposure time: 5000 ms.

Figure 14 depicts selected images from a complete optical sectioning stack of a fibroblast HT29 spheroid. A complete stack is comprised of up to 60 images depending on the size of the spheroid, and every sixth image was chosen for exemplary depiction. All images use the Raman effect and accordingly depict sections of an unstained sample. All pictures were acquired with an illumination power output of 0.93 W/mm^2^ at 660 nm, the AOTF set to 815 nm or 2881 cm^−1^ Raman shift and an exposure time of 5000 ms.

The sections through the cell culture show a light sheet penetration depth of approx. 70 µm. This means that mainly the outer edges can be depicted. The center of the spheroid, however, remains dark due to scattering and absorption events of Raman photons inside the spheroid. Consequently, only in the peripheral areas is illumination of the cross-section possible. The structures shown have a diameter of approx. 5 µm.

**Figure 15:**
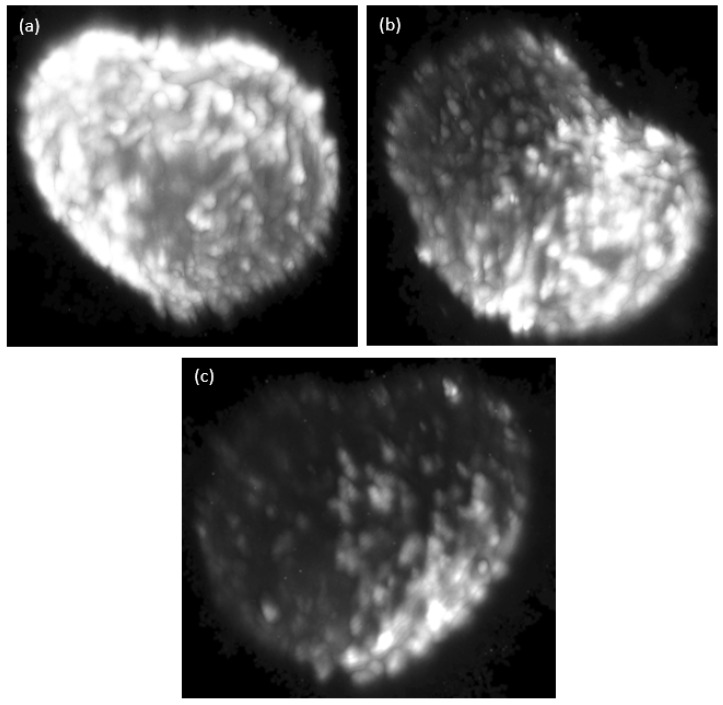
Three-dimensional illustration of the acquired Raman stack from HT29 spheroid from (**a**) 0 degree (front view), (**b**) 90 degrees (side view) and (**c**) 180 degrees (rear view). The stack consists of 52 images. Illuminating wavelength: 660 nm. Optical power output: 0.93 W/mm^2^. AOTF setting: 815 nm or 2881 cm^-1^. Exposure time: 5000 ms.

The image stack acquired through the Raman specification forms the basis to generate a three-dimensional representation. Figure 15 shows the representation of the previously shown stack as a 3D model from different perspectives. The 3D model was rotated at three different angles, specifically at 0 degrees, 90 degrees and 180 degrees. At each of these angles, an image was captured to obtain a frontal view, a side view and a rear view of the sample. This comprehensive approach allowed for a detailed analysis of the sample’s structure and properties from different perspectives.

## 4. Discussion

The proof of concept of the light sheet microscope prototype with the imaging modalities Rayleigh scattering, Raman scattering and fluorescence for generating 3D molecule-specific maps was demonstrated using fibroblast HT29 spheroids.

Both installed lasers are able to optically section spheroid samples in order to acquire image stacks. Each laser generates a light sheet waist of approx. 8 μm thickness resulting in an axial resolution of 8 μm. The lateral resolution was measured at 630 nm per pixel, which is higher by a factor of approx. 12.7 compared to the axial resolution. This must be considered when stacking optical sections to form a 3D model. Nevertheless, the light sheet dimensions facilitate the reliable mapping of spheroids as large as several 100 μm in diameter and successive reconstruction as a 3D model. Furthermore, the light sheet waist defines the minimum *z*-axis increment of the sample positioning system.

The travel accuracy of the positioning system was verified at ±300 nm. Compared to the light sheet thickness of 8 μm, which specifies the smallest step size during sectioning to avoid overlapping of image planes, the accuracy is sufficiently high at 3.75%. To that effect, specifically targeted areas can be positioned with a high repeat accuracy.

Picture repetition stability was also investigated. The results show that the variation of pixel illumination is sufficiently low at a relative range of 1.5% depending on the illumination intensity, thereby guaranteeing a high stability of this parameter as well. This ensures that multiple images at the same sample position carry the same validity. This forms the basis for other image processing methods such as stacking in order to extract further information.

A commercially available Raman spectrometer was used to verify the underlying spectral data for Stokes Raman images. The scanning results show that different information content is provided by each particular modality. The Raman effect allows for marker-free imaging using the high wavenumber region. The penetration depth of 70 µm deteriorates axially when sectioning increasing diameter regions of spheroids. This is likely due to increased absorption of the Raman photons inside the spheroid, resulting in ring shapes around a seemingly void center. The optical sections gained from Draq5 fluorescence show similar detail to the Raman images. However, loss of penetration depth does not affect the fluorescence signal to the same degree as the Raman effect.

A commercially available Raman spectrometer of the type Multispec (Tec5 AG) is used to validate Raman sections by recording the corresponding spectral data. When comparing the spectral data of Raman and fluorescence images, the first impression indicates virtually identical peak positions differing only in intensity. However, the emission spectrum of the fluorescent dye Draq5 ranges from 660 nm to 820 nm or 0 cm^−1^ to 2956 cm^−1^, effectively overlaying the entirety of the Raman spectrum. Accordingly, the bandwidth of the AOTF excises a region of the emission spectrum, resulting in what appears to be identical peak positions. Furthermore, this means that, post-staining, it is impossible to differentiate fluorescence from Raman signals. The fluorescent dye Draq5 was selected to demonstrate that Raman measurements offer a method that yields approximately equivalent results and allows for reliable representation of cell structures stained by the dye. Consequently, the use of Draq5 is considered obsolete for this specific application, leading to reduced sample preparation requirements.

A broader scope of applications and 3D reconstruction of molecule-specific chemical maps will be investigated in future studies in addition to improving image quality.

## 5. Conclusions

Raman-based light sheet imaging of 3D cell cultures is an emerging research field driven by the development of high-quality, custom-built prototypes. The Raman effect provides label-free insight into the molecular composition of samples, fundamentally enabling non-destructive observation of three-dimensional cell cultures, complemented by high-resolution spatial information from Rayleigh scattering. Fluorescent labeling further allows comparison with established imaging techniques. The challenge in device development lies in the extremely weak Raman signal strength, typically orders of magnitude lower than fluorescence effects, themselves hundreds to thousands of times weaker than elastic light scattering [32,33]. To address these challenges, a specialized setup employing wavelength-selective imaging detection has been devised, enabling comprehensive mapping of a specific plane within the cell culture for a particular wavelength range. Increasing the optical output power and reducing the excitation laser wavelength are two parameters that can enhance signal intensity, but careful consideration must be given to avoid compromising the cell samples.

To cover a broad range of molecules, two lasers with different wavelengths are employed, operated alternatively. Wavelength shifting allows for capturing a wide spectral range, and the two images are subsequently merged. This will enable future investigations, for example, of carboxylic acids below 500 wavenumbers and water distributions above 3000 wavenumbers [34,35].

To further enhance the applicability of the microscope, an innovative gel molding sample holder was developed, simplifying sample preparation and mounting while maximizing reproducibility of spatial positioning. Additionally, the sample holder can be individually tailored to match the spheroid diameters using cost-efficient 3D printing. The light sheet dimensions allow for imaging at a cellular scale over a range of several 100 μm, effectively being limited by the field of view of 635 μm by 635 μm, making it suitable for a wide range of 3D cell models.

Acousto-optic tunable filters (AOTFs) enable rapid wavelength switching, allowing for the acquisition of images with different wavelength ranges in quick succession. The speed of image acquisition is limited by camera sensitivity, and efforts are being made to fully utilize current technological capabilities. Currently, a high-sensitivity 16-bit camera is installed, but future applications may include the use of image intensifiers to further improve performance and more effectively capture the weak Raman signal strength. Another improvement could involve enabling discrimination between different molecules, particularly distinguishing between various types of collagens [36].

The choice of microscope objectives in light sheet microscopy is derived from the desired field of view reaching up to 0.6 mm [13]. Increasing the magnification of the objectives may, in theory, improve the optical resolution in order to reveal smaller details. However, there are inherent limitations to improving resolution solely through higher magnification [37]. Light sheet microscopy is subject to diffraction effects, scattering in the sample and phototoxicity. Diffraction of light occurs when it passes through a narrow aperture such as the light sheet, resulting in limited resolution dictated by the Abbe limit. Higher magnification alone cannot overcome these diffraction effects. Light scattering within the sample can also contribute to blurring and reduced resolution, particularly in thicker or highly scattering samples. Higher magnification may exacerbate scattering, leading to decreased contrast and poorer resolution. Moreover, phototoxic effects can occur when the sample is exposed to intense light, and higher magnification typically entails higher light intensity at the sample, potentially increasing the risk of phototoxic reactions. This can compromise cell viability and lead to artifacts. Overall, there are constraints on improving optical resolution through the use of higher magnification objectives in this setup. An optimized selection of magnification, in conjunction with appropriate illumination techniques and image processing, is crucial to achieve optimal results.

Currently, the device allows for the observation of molecular compounds between 2700 and 3000 cm^−1^, thereby generating information about distribution of collagen, for instance. When combined with conventional elastic 3D imaging techniques such as reflection tomography, external structures of cells, such as flagella, can also be recognized [38]. However, distinguishing between different peptides, such as various types of collagens, is currently not possible. In comparison, alternative variants of state-of-the-art light sheet Raman micro-spectroscopy allow for determining lipid deposition and consumption, local cell density and water content, for example, in zebra fish embryos or fungi [14,39]. However, a combination with multiple inelastic light measuring methods is not provided in a single device. Future applications could also involve the investigation of deuterated objects, as the Raman vibrations of deuterated hydrocarbons are shifted to even higher wavenumbers, around 4000 cm^−1^.

In summary, Raman-based light sheet imaging of 3D cell cultures offers significant potential for exploring the molecular composition and behavior of cells in a three-dimensional environment. The ability to non-destructively observe three-dimensional cell cultures and obtain molecular information can yield new insights into drug effects, nutrition, genetic disorders and other aspects of cell growth and metabolism. In particular, the development of organoids, three-dimensional tissue-like structures, opens up new possibilities for studying complex cell cultures [40]. Future work will involve further evaluation of the multi-modal light sheet microscope with larger sample quantities and different cell models. Additional optimizations and advancements of the device will be pursued to improve the performance and versatility of 3D cell culture imaging. 

## Figures and Tables

**Table 1:** Component list as numbered in Figure 2, Figure 3, Figure 4 and Figure 5.

No.	Specification	Manufacturer
1	LuxX Laser 785 nm, adjustable laser power 0.5–200 mW	Omicron Laserage GmbH (Rodgau, Germany)
2	LuxX Laser 660 nm, adjustable laser power 0.5–130 mW	Omicron Laserage GmbH
3	Broadband mirror, Ø25.4 mm, EO2-coated mounted in Polaris K1 Kinematic Mirror Mount	Thorlabs GmbH (Newton, NJ, USA)
4	Broadband mirror, Ø25.4 mm, EO3-coated mounted in Polaris K1 Kinematic Mirror Mount	Thorlabs GmbH
5	Broadband mirror, Ø25.4 mm, EO2-coated mounted in Polaris K1 Kinematic Mirror Mount	Thorlabs GmbH
6	Broadband mirror, Ø25.4 mm, EO3-coated mounted in Polaris K1 Kinematic Mirror Mount	Thorlabs GmbH
7	BrightLine laser dichroic beamsplitter, 25 mm × 36 mm, reflection band 350–671 nm, transmission band 702–1200 nm	Semrock (Rochester, NY, USA)
8	Broadband mirror, Ø25.4 mm, EO2-coated mounted in Polaris K1 Kinematic Mirror Mount	Thorlabs GmbH
9	Mounted achromatic doublet lens, Ø12.7 mm, focal length 25 mm, anti-reflex coating 400–1100 nm	Thorlabs GmbH
10	Mounted achromatic doublet lens, Ø12.7 mm, focal length 50 mm, anti-reflex coating 400–1100 nm	Thorlabs GmbH
11	Mounted cylindrical achromatic doublet lens, Ø25.4 mm, focal length 50 mm, anti-reflex coating 650–1050 nm	Thorlabs GmbH
12	UMPLFLN10XW water dipping objective, magnification 10×, numerical aperture 0.3, working distance 3.5 mm, semi-apochromat	Evident Europe GmbH (Hamburg, Germany)
13	UMPLFLN20XW water dipping objective, magnification 20×, numerical aperture 0.5, working distance 3.5 mm, semi-apochromat	Evident
14	Acousto-optic tunable filter, spectral range 550–1000 nm	Brimrose (Sparks Glencoe, MD, USA)
15	Polarization filter	Thorlabs GmbH
16	6 position motorized filter wheel	Thorlabs GmbH
17	Longpass filter 660 nm	Semrock
18	Notch filter 660 nm	Semrock
19	Shortpass filter 660 nm	Semrock
20	Longpass filter 785 nm	Semrock
21a	Tube lens U-TLU and C-mount (U-TV0.5XC-3)	Evident
21b	Aspheric condenser lens, Ø25 mm, focal length 20 mm, anti-reflex coating 650–1050 nm	Thorlabs GmbH
22a	sCMOS camera ORCA Flash 4.0 LT+	Hamamatsu (Hamamatsu-shi, Japan)
22b	CXY1 two-axis translating lens mount, Ø550 μm optic fiber	Thorlabs GmbH
23	USB-4D-stage (X, Y, Z, R)	Picard-Industries (Orleans, NY, USA)
24	Sample chamber, aluminum mounting frame, acrylic water chamber	CeMOS (Mannheim, Germany)
25	MultiSpec^®^ Raman spectrometer	tec5 GmbH (Steinbach, Germany)

**Table 2:** Workflow and setup parameters.

Scan No.	Modality	Incident Wavelength	Optical Power	Integration Time	Detected Wavelength	Spheroid Condition	Detecting Device
1 *	Rayleigh scattering	785 nm	1 mW	100 ms	785 nm	PFA-fixed, mounted in 2% agarose, unstained	ORCA Flash 4.0 LT+ Hamamatsu (Hamamatsu-shi, Japan)
2		785 nm			785 nm		
3		660 nm			660 nm		
4	Stokes scattering	660 nm	130 mW	5000 ms	817 nm		
5		785 nm	200 mW	5000 ms			
6	Stokes scattering	660 nm	130 mW	5000 ms	817 nm	PFA-fixed, mounted in 2% agarose, unstained	MultiSpec^®^ Raman spectrometer tec5 GmbH (Steinbach, Germany)
7		785 nm	200 mW	5000 ms			
8 *	Rayleigh scattering	785 nm	1 mW	100 ms	785 nm	PFA-fixed, mounted in 2% agarose, Draq5-stained	ORCA Flash 4.0 LT+
9		785 nm	1 mW	100 ms	785 nm		
10		660 nm	1 mW	100 ms	660 nm		
11	Fluorescence marker	660 nm				PFA-fixed, mounted in 2% agarose, Draq5-stained	ORCA Flash 4.0 LT+

* Manual determination of spatial scan boundaries according to spheroid diameter.

**Table 3:** Quartiles and arithmetic mean of the travel distance per step for each linear axis. Calculated from a total of 80 experimental runs per axis.

Axis	Q0 (Minimum)/μm	Q1 /μm	Q2 (Median)/μm	Q3/μm	Q4 (Maximum)/μm	Mean/μm
X	0.90	1.33	1.50	1.60	2.00	1.47
Y	1.10	1.39	1.50	1.60	1.86	1.48
Z	1.15	1.40	1.50	1.58	1.83	1.48
Y Extension	0.55	0.80	0.95	1.10	1.40	0.96

**Table 4:** Image stability of 36 pixels chosen at random. Calculated from two image sets of 10 images each at 1 mW and 130 mW illuminating power. Relative signal variation of all pixels is displayed as quartiles of 360 values.

Illuminating Optical Power/mW	Q0 (Minimum)/%	Q1 /%	Q2 (Median)/%	Q3/%	Q4 (Maximum)/%
1	−26.15	−3.72	−0.18	3.83	75.80
130	−16.07	−4.20	−0.24	4.48	15.38

**Table 5:** Raman peak positions and vibrational modes.

Peak Position/cm^−1^	Vibrational Mode	References
2850–2860	CH_2_ symmetric stretching	[28]
2880–2895	CH_2_ asymmetric stretching	[29]
2929–2937	CH_3_ stretching	[30]

## Data Availability

The data presented in this study are available in supplementary material.

## Supplementary Materials

The following supporting information can be downloaded at: https://www.mdpi.com/article/10.3390/mi14091739/s1, Figure S1. Measured travel distance in μm per step calculated from 10 step increments. The dotted line indicates the mean value of the step size. Figure S2. Relative deviation of step sizes from the mean step size in %. Red bars indicate the mean deviation of each increment for a total of three measurement sets. Figure S3. Image coordinates of investigated pixels for the determination of the signal fluctuation of the camera chip with (a) Rayleigh and (b) Raman. The spheroid was illuminated at 660 nm emission and detected at 670 nm with a bandwidth of 3 nm. The image was cropped from its original size to improve visibility of the examined areas. Figure S4. Relative intensity deviation from the mean value of 10 measurements of nine individual pixels at position 1 for Rayleigh. Figure S5. Relative intensity deviation from the mean value of 10 measurements of nine individual pixels at position 2 for Rayleigh. Figure S6. Relative intensity deviation from the mean value of 10 measurements of nine individual pixels at position 3 for Rayleigh. Figure S7. Relative intensity deviation from the mean value of 10 measurements of nine individual pixels at position 1 for Raman. Figure S8. Relative intensity deviation from the mean value of 10 measurements of nine individual pixels at position 2 for Raman. Figure S9. Relative intensity deviation from the mean value of 10 measurements of nine individual pixels at position 3 for Raman.

## References

[1] Macleod M., Michie S., Roberts I., Dirnagl U., Chalmers I., Ioannidis J., Salman R., Chan A., Glasziou P. (2014). Biomedical reseach: Increasing value, reducing waste. Lancet.

[2] Yu J., Navarro J., Coburn J., Mahadik B., Molnar J., Holmes J., Nam A., Fisher J. (2019). Current and Future Perspective on Skin Tissue Engineering: Key Features of Biomedical Research, Translational Assessment, and Clinical Application. Adv. Healthc. Mater..

[3] Musumeci G. (2014). Past, present and future: Overview on Histology and histopathology. J. Histol. Histopathol..

[4] Mazzarini M., Falchi M., Bani D., Migliaccio A. (2020). Evolution and new frontiers of histology in bio-medical research. Microsc. Res. Tech..

[5] Ader M., Tanaka E. (2014). Modeling human development in 3D culture. Curr. Opin. Cell Biol..

[6] Park Y., Huh K., Kang S.-W. (2021). Applications of Biomaterials in 3D Cell Culture and Contribution of 3D Cell Culture to Drug Development and Basic Biomedical Research. Int. J. Mol. Sci..

[7] Duval K., Grover H., Han L.-H., Mou Y., Pegoraro A., Fredberg J., Chen Z. (2017). Modeling Physiological Events in 2D vs. 3D Cell Culture. Physiology.

[8] Ravi M., Paramesh V., Kaviya S.R., Anuradha E., Solomon F.D. (2014). 3D Cell Culture Systems: Advantages and Applications. J. Cell. Physiol..

[9] Knight E., Przyborski S. (2014). Advances in 3D cell cultures technologies enabling tissue-like structures to be created in vitro. J. Anat..

[10] Pampaloni F., Chang B.-J., Stelzer E. (2015). Light sheet-based fluorescence microscopy (LSFM) for the quantitative imaging of cells and tissues. Cell Tissue Res..

[11] Olarte O., Andilla J., Gualda E., Loza-Alvarez P. (2018). Light-sheet microscopy: A tutorial. Adv. Opt. Photonics.

[12] Girkin J., Carvalho M. (2018). The light-sheet microscopy revolution. J. Opt..

[13] Weber M., Mickoleit M., Huisken J. (2014). Chapter 11—Light sheet microscopy. Methods Cell Biol..

[14] Mueller W., Kielhorn M., Schmitt M., Popp J., Heintzmann R. (2016). Light Sheet Raman micro-spectroscopy. Optica.

[15] Miles R., Lempert W., Forkey J. (2001). Laser Rayleigh scattering. Meas. Sci. Technol..

[16] Koenigstein J. (1972). Introduction to the Theory of the Raman Effect.

[17] Eberhardt K., Stiebing C., Matthäus C., Schmitt M., Popp J. (2015). Advantages and limitations of Raman spectroscopy for molecular diagnostics: An update. Expert Rev. Mol. Diagn..

[18] Oshima Y., Sato H., Kajiura-Kobayashi H., Kimura T., Naruse K., Nonaka S. (2012). Light sheet-excited spontaneous Raman imaging of a living fish by optical sectioning in a wide field Raman microscope. Opt. Express.

[19] Rocha-Mendoza I., Licea-Rodriguez J., Marro M., Olarte O., Plata-Sanchez M., Loza-Alvarez P. (2015). Rapid spontaneous Raman light sheet microscopy using cw-lasers and tunable filters. Biomed. Opt. Express.

[20] Cox A., DeWeerd A., Linden J. (2002). An experiment to measure Mie and Rayleigh total scattering cross sections. Am. J. Phys..

[21] Pully V., Lenferink A., Otto C. (2009). Hybrid Rayleigh, Raman and two-photon excited fluorescence spectral confocal microscopy of living cells. J. Raman Spectrosc..

[22] Di Battista D., Merino D., Zacharakis G., Loza-Alvarez P., Olarte O. (2019). Enhanced Light Sheet Elastic Scattering Microscopy by Using a Supercontinuum Laser. Methods Protoc..

[23] Pitrone P., Schindelin J., Stuyvenberg L., Preibisch S., Weber M., Eliceiri K., Huisken J., Tomancak P. (2013). OpenSPIM: An open-access light-sheet microscopy platform. Nat. Methods.

[24] Brimrose Corp Technical Specifications Free Space AOTF. https://www.brimrose.com/free-space-ao/acousto-optic-tunable-filters.

[25] Pudlas M. (2012). Nicht Invasive Diagnostik in der Regenerativen Medizin mittels Raman-Spektroskopie.

[26] Smith P., Wiltshire M., Errington R. (2004). DRAQ5 Labeling of Nuclear DNA in Live and Fixed Cells. Curr. Protoc. Cytom..

[27] Savitzky A., Golay M. (1964). Smoothing and Differentation of Data by Simplified Least Square Procedures. Anal. Chem..

[28] Slatinskaya O., Luneva O., Deev L., Orlov A., Maksimov G. (2020). Conformational Changes that occur in Heme and Globin upon Temperature Variations and Normobaric Hypocia. Mol. Biophys..

[29] Peike C., Kaltenbach T., Weiß K.-A., Koehl M. (2011). Non-destructive degradation analysis of encapsulants in PV modules by Raman Spectroscopy. Sol. Energy Mater. Sol. Cells.

[30] Hill I., Levin I. (1979). Vibrational spectra and carbonhydrogen stretching mode assignments for a series of n-alkyl carboxylic acids. J. Chem. Phys..

[31] Dobrucki J.W., Kubitscheck U., Kubitscheck U. (2017). Fluorescence Microscopy.

[32] Sivaprakasam V., Dennis K. (2003). Effect of polarization and geometric factors on quantitative laser-induced fluorescence- to-Raman intensity ratios of water samples and a new calibration technique. J. Opt. Soc. Am. B.

[33] Manninen S., Pitkänen T., Koikkalainen S., Paakkari T. (1984). Study of the ratio of elastic to inelastic scattering of photons. Int. J. Appl. Radiat. Isot..

[34] Génin F., Quilès F., Burneau A. (2001). Infrared and Raman spectroscopic study of carboxylic acids in heavy water. Phys. Chem. Chem. Phys..

[35] Cross P., Burnham J., Leighton P. (1937). The Raman Spectrum and the Structure of Water. J. Am. Chem. Soc..

[36] Gullekson C., Lucas L., Hewitt K., Kreplak K. (2011). Sufrace-Sensitive Raman Spectroscopy of Collagen I Fibrils. Biophys. J..

[37] Xiaodong C., Bin Z., Hong L. (2011). Optical and digital microscopic imaging techniques and applications in pathology. Anal. Cell. Pathol..

[38] Nicastro D., McIntosh J., Baumeister W. (2005). 3D structure of eukaryotic flagella in a quiescent state revealed by cryo-electron tomography. Proc. Natl. Acad. Sci. USA.

[39] Siddhanta S., Paidi S., Bushley K., Prasad R. (2017). Exploring Morphological and Biochemical Linkages in Fungal Growth with Label-Free Light Sheet Microscopy and Raman Spectroscopy. Chem. Phys. Chem..

[40] Miao S., Liu A., Yang X., Gong J., Yu M., Yao X., Wang H., He Y. (2021). 3D Cell Culture—Can It Be as Popular as 2D Cell Culture?. Adv. Biomed. Res..

