# Supplementary material for: Innovative Imaging Techniques: A Conceptual Exploration of Multi-Modal Raman Light Sheet Microscopy
Source: Micromachines (Basel). 2023 Sep 5;14(9):1739. doi: 10.3390/mi14091739 (PMC10536344; doi:10.3390/mi14091739)
Supplement: Supplementary file 1 [file micromachines-14-01739-s001.zip › micromachines-2522836-supplementary.pdf]

Supplementary Materials for:

Innovative Imaging Techniques: A Conceptual Exploration of Multi-Modal Raman Light Sheet Microscopy

Steffen Manser[1,†], Shaun Keck[1,†], Mario Vitacolonna[1], Felix Wuehler[1], Ruediger Rudolf[1], Matthias Raedle[1,*]

[1] Center for Mass Spectrometry and Optical Spectroscopy (CeMOS), University of Applied Science Mannheim, 68163 Mannheim, Baden-Württemberg, Germany

[†] These authors contributed equally to this work

* Correspondence: m.raedle@hs-mannheim.de

This supplementary material presents a comprehensive analysis of the traversing unit's accuracy in reaching the desired locations. It conducts a detailed examination of the distribution of pixel intensities and the resulting precision in capturing individual pixel values. Additionally, upon request, the complete set of image data and spectra corresponding to the individual stacks of the cell culture can be obtained from the corresponding author.

Travel Distance

To compensate for mechanical backlash, the travel distance is traversed three times consecutively in the same direction. It is noteworthy that the origin for each drive remains consistent across all measurements. When resetting the origin, it is intentionally passed over, and subsequent measurements commence in the direction of travel. To provide a representative measure, the average travel distance per step is computed from the dataset. Figure S1 illustrates both the resulting average value and the variation observed in individual measurements.

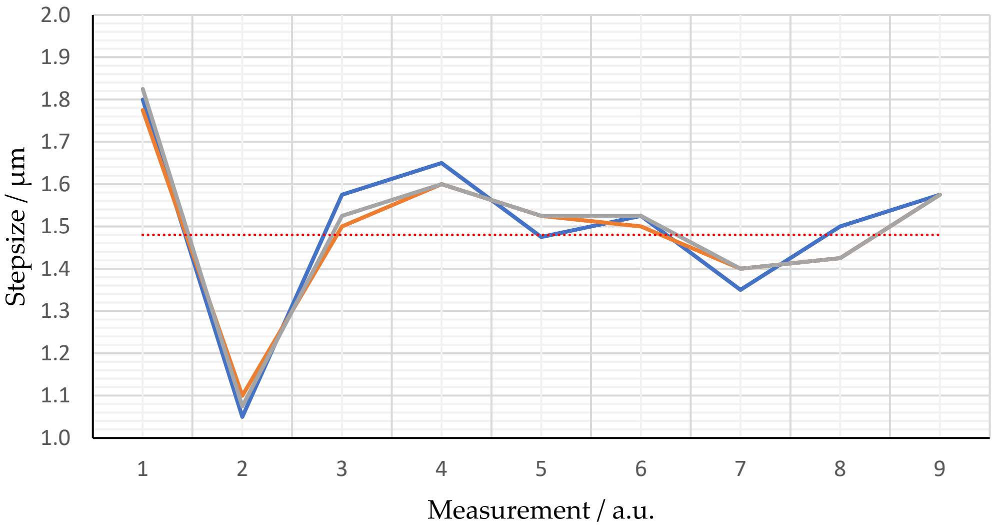

Figure S1. Measured travel distance in µm per step calculated from 10 step increments. The dotted line indicates the mean value of the step size.

The average travel distance per step is calculated to be 1.47 µm, establishing a baseline for assessing the variation in step size relative to this mean value. To provide a comprehensive overview of the step size variation across all measurement sets, Figure S2 presents a detailed summary. The red bars in the figure represent the average deviation of individual measurement sets, providing valuable insights into both the consistency and dispersion of the step sizes.

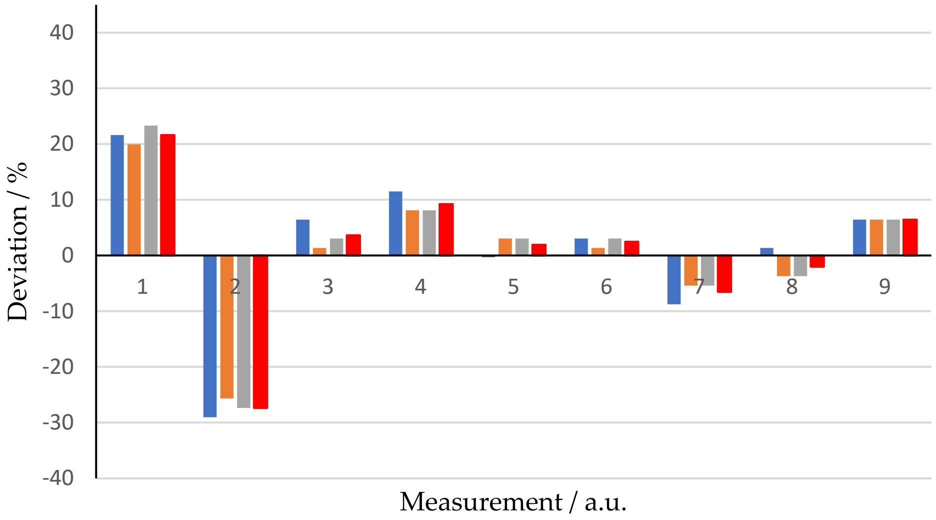

Figure S2. Relative deviation of step sizes from the mean step size in %. Red bars indicate the mean deviation of each increment for a total of three measurement sets.

The mean relative accuracy of the positioning system is observed to range from -28% to 22% when compared to the calculated average step size. This range indicates the variation in the positioning system's ability to achieve accurate and precise step sizes relative to the expected average value.

Pixel Intensity

To examine the range of signal intensity variation in imaging, two sets of images, each consisting of ten pictures of a single spheroid section, were captured and their pixel values compared. In the first image set, referred to as "Rayleigh," the spheroid was illuminated with an emission wavelength of 660 nm at an optical power output of 1 mW. To avoid overlapping the image with the exciting wavelength and to eliminate signal intensity from the blocking filters, the Acousto-Optic Tunable Filter (AOTF) was set to 670 nm. The second image set, referred to as "Raman," was acquired at an optical power output of 130 mW, with the AOTF set to 815 nm (2800 cm^{-1}). In Figure S3, the specific areas of interest used for the comparative analysis are highlighted in a sample image from the measurement set, providing a visual reference for the regions under investigation.

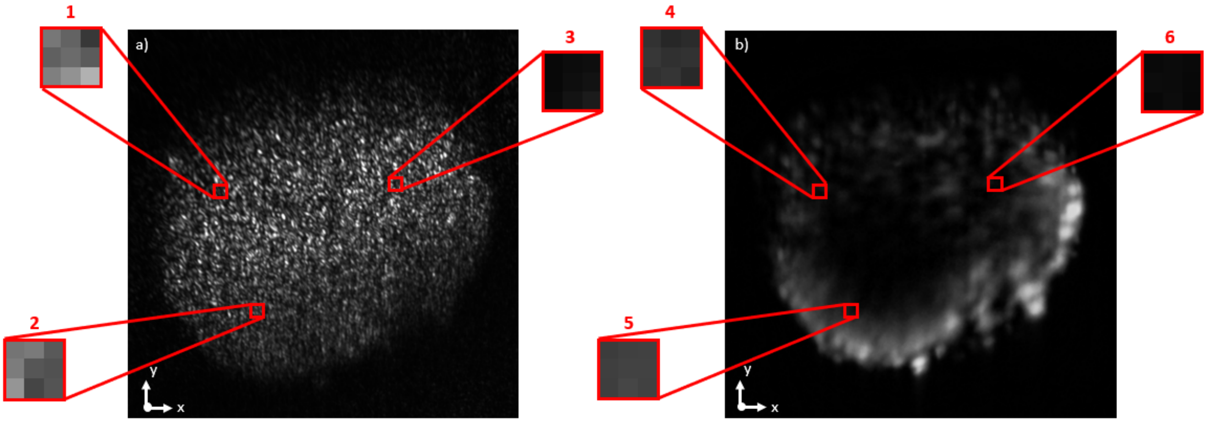

Figure S3. Image coordinates of investigated pixels for the determination of the signal fluctuation of the camera chip with a) Rayleigh and b) Raman. The spheroid was illuminated at 660 nm emission and detected at 670 nm with a bandwidth of 3 nm. The image was cropped from its original size to improve visibility of the examined areas.

Figure S4 depicts the relative deviation, expressed as a percentage, of individual pixel intensities from the arithmetic mean of all 10 measurements. This visualization provides insights into the variation of pixel intensities across the image set, highlighting the deviations from the average intensity value.

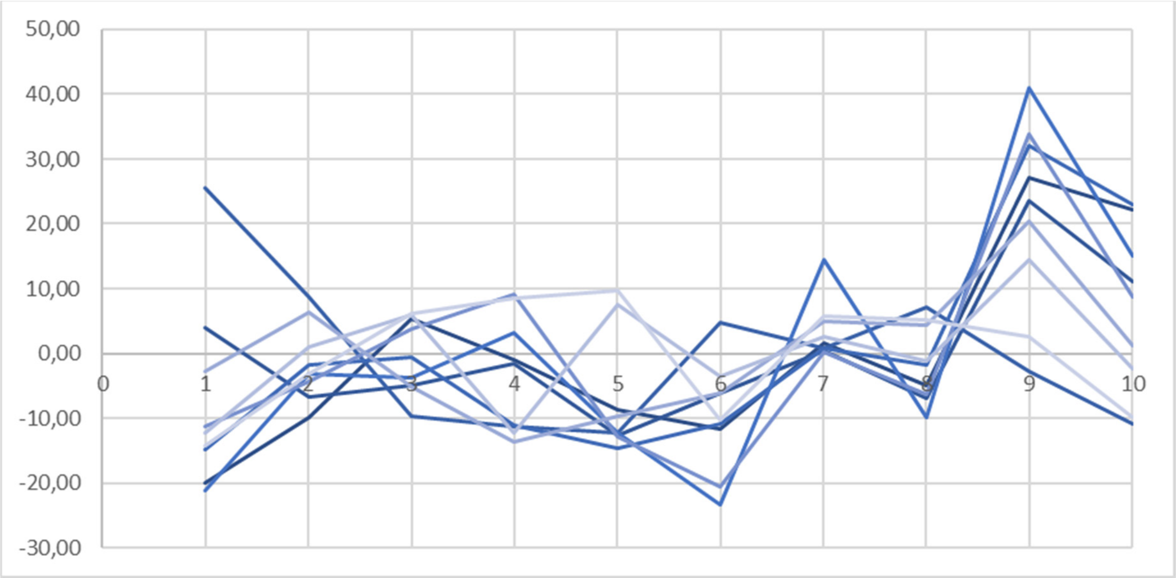

Figure S4. Relative intensity deviation from the mean value of 10 measurements of nine individual pixels at position 1 for Rayleigh.

Pixel 1 displays a noteworthy intensity variation ranging from approximately -24% to +40% relative to the mean value calculated from the intensities of 10 pixels. This data indicates a relatively significant fluctuation in the intensity of Pixel 1. In the subsequent analysis, we delve deeper into the relative fluctuation of Pixel 2 and present a concise summary in Figure S5. This visual representation offers a comprehensive depiction of the observed variations in the intensity of Pixel 2, enabling a more profound comprehension of its behavior in relation to the mean intensity value.

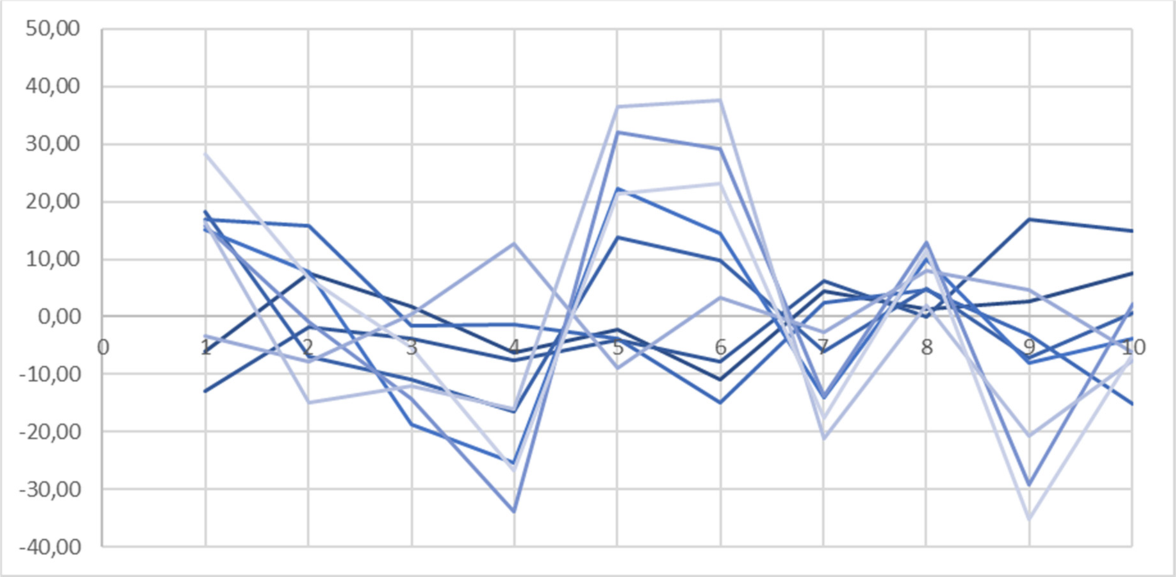

Figure S5. Relative intensity deviation from the mean value of 10 measurements of nine individual pixels at position 2 for Rayleigh.

The signal intensities at pixel position 2 for Rayleigh exhibit a deviation range of -33% to +38% of the 10 measurements.

Shifting focus to the third investigated pixel location, Figure S6 provides a detailed representation of the relative signal fluctuations at this specific location. By examining this figure, one can gain a more comprehensive understanding of the intensity variations observed for Pixel 3.

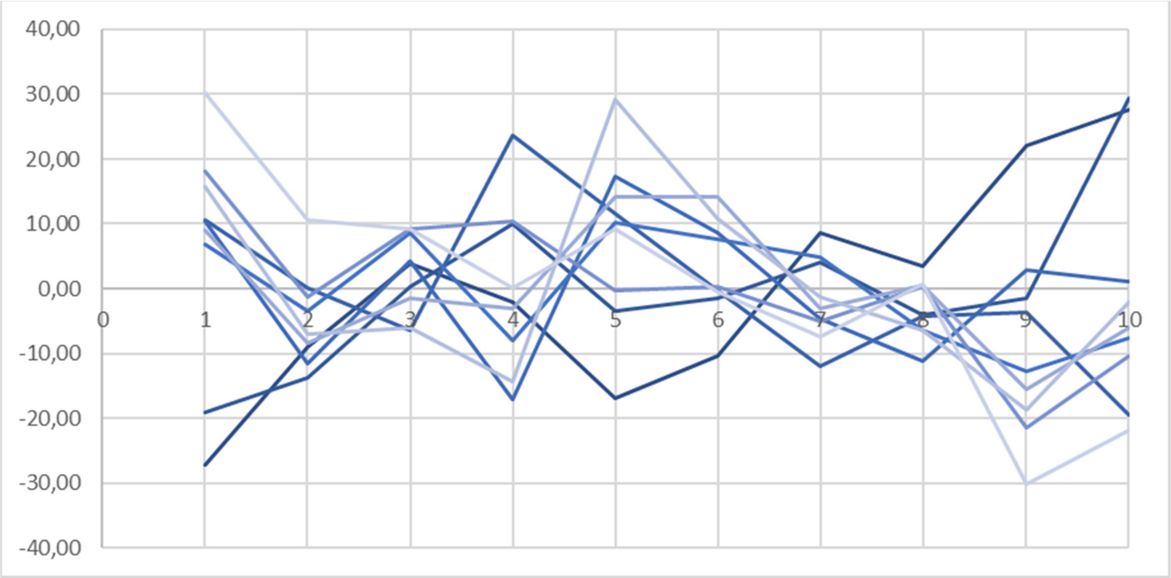

Figure S6. Relative intensity deviation from the mean value of 10 measurements of nine individual pixels at position 3 for Rayleigh.

The signal intensity at pixel location 3 exhibits variations ranging from -30% to +30% relative to the mean calculated signal intensity. These measurement sets serve as the foundation for calibrating pixel values, thereby enhancing the comparability of individual measurements.

For the Raman-Images the results are shown on the following Figures. Figure S7 shows the results for the measurement at pixel position 1.

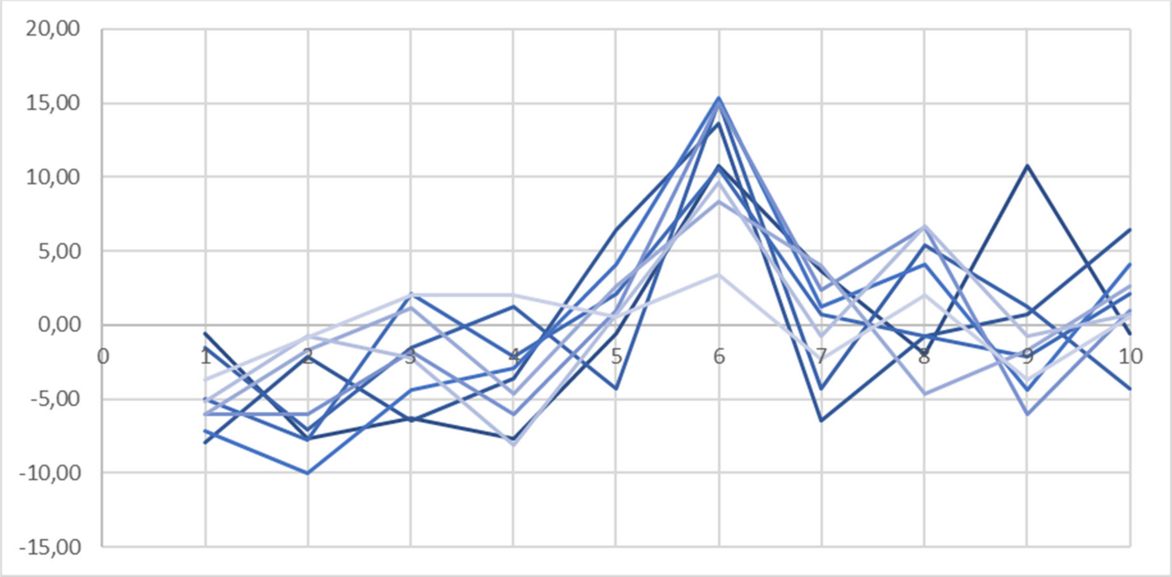

Figure S7. Relative intensity deviation from the mean value of 10 measurements of nine individual pixels at position 1 for Raman.

The signal intensities at pixel position 1 for Raman exhibit a deviation range of -10% to +15% of the 10 measurements.

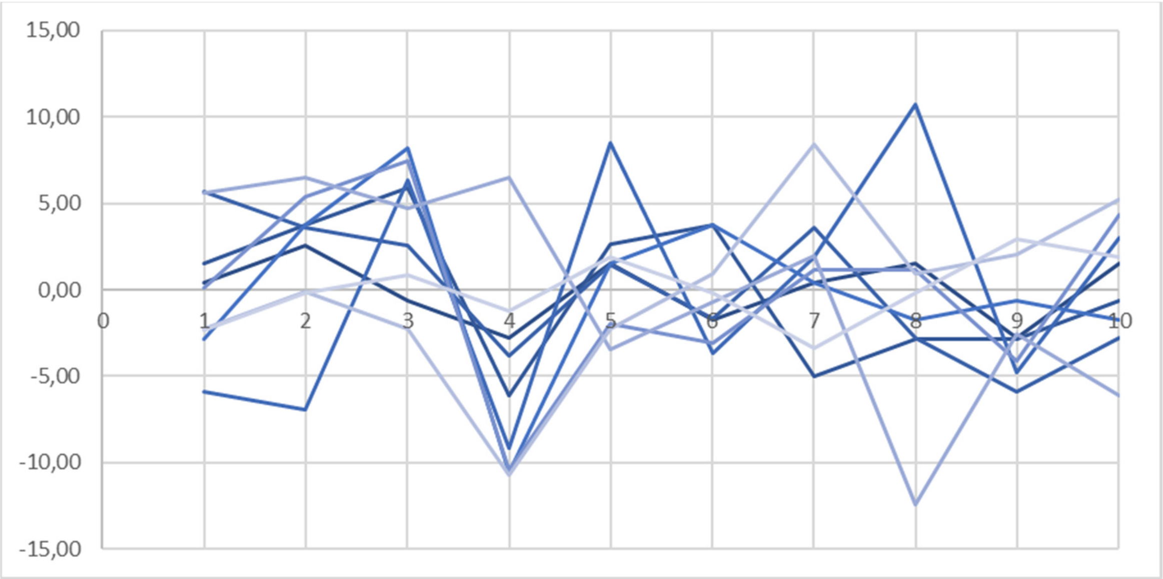

Figure S8. Relative intensity deviation from the mean value of 10 measurements of nine individual pixels at position 2 for Raman.

The signal intensities at pixel position 3 for Raman exhibit a deviation range of -12% to +11% of the 10 measurements.

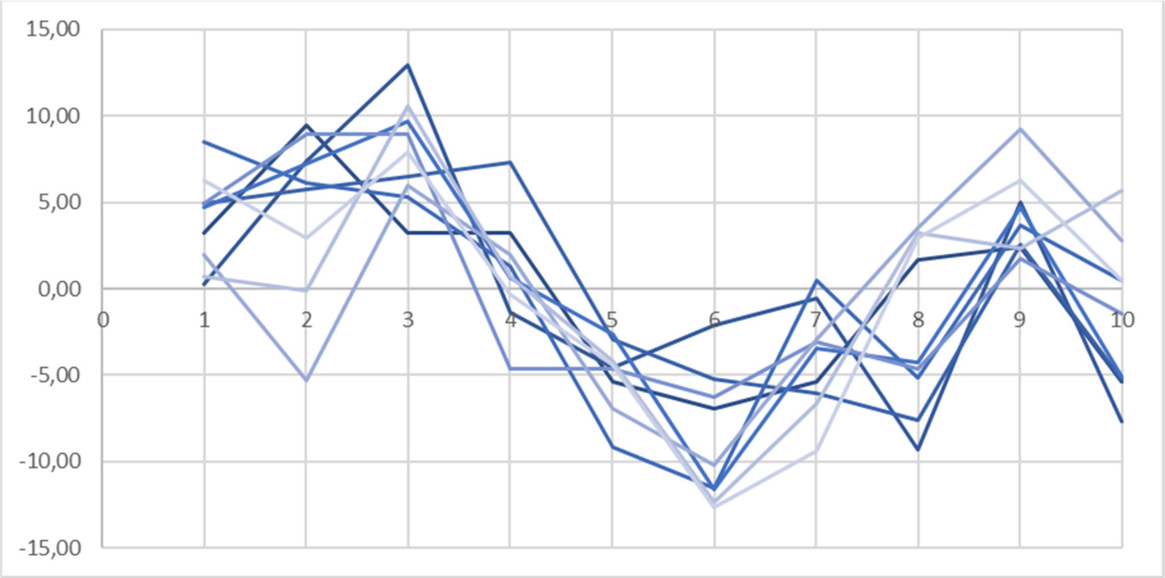

Figure S9. Relative intensity deviation from the mean value of 10 measurements of nine individual pixels at position 3 for Raman.

The signal intensities at pixel position 3 for Raman exhibit a deviation range of -13% to +13% of the 10 measurements.
